# Left paraduodenal hernia – A diagnostic challenge: Case report

**DOI:** 10.1016/j.ijscr.2021.106138

**Published:** 2021-06-29

**Authors:** Dragan Manojlović, Nenad Čekić, Mario Palinkaš

**Affiliations:** a National Memorial Hospital Vukovar, Department of Surgery, Croatia; b Faculty of Medicine, Josip Juraj Strossmayer University of Osijek, Croatia; c Faculty of Dental Medicine and Health, Josip Juraj Strossmayer University of Osijek, Croatia

**Keywords:** Internal hernia, Paraduodenal hernia, Case report

## Abstract

**Introduction and importance:**

Internal hernias represent the penetration of intestinal loops through congenital or acquired openings within the mesentery or peritoneum. One such hernia is the paraduodenal hernia, which is the most common type of internal hernia (53%) (Yun et al., 2010) [1]. Due to the variable and non-specific clinical presentation, it is difficult to make a correct diagnosis. Preoperative computed tomography of the abdomen facilitates diagnosis and timely surgical intervention, which can be performed openly or laparoscopically (Coakley et al., 2012) [2].

**Case presentation:**

In this paper, the case of a 39-year-old patient with left paraduodenal hernia will be presented. He arrived in the emergency department with sudden onset of abdominal pain. The diagnosis was established using computed tomography and an open surgical procedure was successfully performed.

**Discussion:**

Clinical presentation of paraduodenal hernia ranges from asymptomatic to manifest. The greatest difficulty regarding management of paraduodenal hernias lies in their diagnosis. Many studies have shown that the best option for diagnosis is computed tomography (CT). Open and laparoscopic techniques are used in treatment of paraduodenal hernias with similar results.

**Conclusion:**

The case of a 39-year-old male patient with LPDH with non-specific symptomatology was presented. CT scan is the best diagnostic option for this condition. Open surgical approach was used with great success.

## Highlights

• Internal hernias represent the penetration of intestinal loops within the mesentery.
• Most common internal hernias are paraduodenal hernias.
• Best diagnostic tool for internal hernias is computed tomography.
• Surgical options for internal hernias include open and laparoscopic approach.

## 1 Introduction

An internal hernia can be defined as the protrusion of parts of internal organs (most commonly the meander of the small intestine) through normal (foramen of Winslow), paranormal (ileocecal, supravesical, paraduodenal fossa) or abnormal (transomental) mesenteric or peritoneal defects into various sections of either the abdominal or the pelvic cavity [[1], [2], [3]]. These defects can be acquired (caused by abdominal surgery, trauma, peritoneal infection or ischemic processes, increase in intra-abdominal pressure and consequent dilatation of the Winslow opening, omental atrophy) or congenital (embryonic malformations such as intestinal malrotation, absence of retroperitoneal attachments – ileocecal fossa, paraduodenal fossa).

Internal hernias are rare and pose a significant diagnostic and therapeutic challenge for both radiologists and clinicians [4]. Paraduodenal hernia is the most common form of internal hernia and occurs with an incidence of 53% of all internal hernias; it causes 0.2–0.9% of all cases of intestinal obstruction [3]. In regard to gender, internal hernias occur 3 times more often in men than in women.

There are two types of paraduodenal hernias: left-sided (75% of all PDHs) and right-sided (25% of all PDHs) [4]. Clinical presentation of paraduodenal hernia varies from asymptomatic through non-specific symptoms, such as digestive disorders and chronic abdominal pain, to symptoms of intestinal obstruction, which can, in case of misdiagnosis, lead to intestinal perforation and endanger the patient's life [4]. The diagnosis is made based on medical history, clinical examination of the patient, laboratory diagnostics, and preoperative computed tomography. Despite the stated diagnostic possibilities, the final diagnosis is often made during surgery.

Treatment consists of surgery, either in the form of open surgery or laparoscopy. Laparoscopic surgery is preferred by experienced surgeons in high volume centers. Recovery after the laparoscopic procedure is faster, but long-term outcomes are similar for both methods [5]. In this paper, we will describe the case of left paraduodenal hernia in a 39-year-old man who presented with acute abdominal pain on arrival at the hospital, and who was diagnosed using abdominal CT and successfully treated with open surgery. The work has been reported in line with the SCARE criteria [6].

## 2 Classification

There are numerous classifications of internal hernias, and one of the more acceptable ones is suggested by Welch, who divides internal hernias into 8 types: 1a: left paraduodenal hernia, 1b: right paraduodenal hernia, 2: foramen of Winslow hernia, 3: pericecal hernia; 4: sigmoid mesocolon-related hernia; 5: transmesenteric hernia; 6: transomental hernia; 7: supravesical and pelvic hernia. Doishita et al. classify internal hernias into 3 main groups according to the type of hernia opening (normal opening, recess into retroperitoneum or unusual peritoneal fossa, and abnormal opening in mesentery or peritoneal ligament) [7].

Left paraduodenal hernias (LPDHs) occur when the proximal jejunum or parts of the duodenum prolapse through the paraduodenal fossa or Landzert's fossa [6]. This is an opening at the duodenojejunal junction (the junction of the transverse mesocolon, the descending mesocolon, and the mesentery of the small intestine), behind the descending mesocolon, and to the left of the fourth segment of the duodenum [6]. Pathoanatomically, in LPDH, small bowel loops enter posteroinferiorly through the mesocolic defect and remain trapped in Landzert's fossa, further spreading into the descending mesocolon and the left half of the transverse mesocolon. The afferent gyrus enters the hernia sac posteriorly, at the point where the duodenum emerges from its fixed retroperitoneal position, so that only the efferent gyrus passes through the hernia opening [8], [9], [10].

## 3 Case presentation

Written informed consent was obtained from the patient for publication of this case report and the accompanying images. A copy of the written consent is available for review by the Editor-in-Chief of this journal upon request.

A 39-year-old patient came to the emergency surgical department in the district general hospital in 2020, with the clinical picture of spasmodic pain in the epigastrium, with suprapubic propagation in the left back. The patient vomited 5 to 6 times. He took a tablet of ibuprofen, after which the abdominal pain intensified and he vomited a few more times. He was drenched in cold sweat and felt faint. He had a normal stool and did not complain about any urinary problems. He did not suffer from any serious illnesses. He consumed gastroprotective drugs, had appendix surgery, and multiple lipomas of the anterior abdominal wall. The medical history showed that he had experienced similar abdominal pain in the past, since childhood, with a weakened appetite and occasional nausea and vomiting after eating. In the past, he was treated conservatively, without an accurate and definitive diagnosis. He last underwent a gastroscopy 3 years ago, when he was diagnosed with chronic gastritis and gastroesophageal reflux disease (GERD). On that occasion, a biopsy and pathohistological diagnosis were performed – active chronic gastritis, Helicobacter pylori – positive. Eradication therapy was performed. The patient did not smoke, drank alcohol occasionally, and denied any drug allergies. In regard to drugs, he took pantoprazole 40 mg once a day and ibuprofen as needed.

*Status praesens*: conscious, in contact, oriented, eupneic. Skin and visible mucous membranes normal. *Cor*: rhythmic action, clear tones, no murmurs. Arterial blood pressure on both arms 140/80 mmHg. *Lung*: right basal, impaired respiratory noise. *Abdomen*: meteoric, soft, tender to deeper palpation in the epigastrium and suprapubic, without organomegaly, peristalsis weakened. Lumbar succussion negative, bilateral. *Limbs*: no edema, normal peripheral arterial pulsations. *Digitorectally*: ampoule filled with feces, no neoplasm is felt at the fingertips, normal colored stool on the glove. Laboratory findings are unobtrusive, except for leukocytosis of 11.4 × 10^9^/L.

**Fig. 1:**
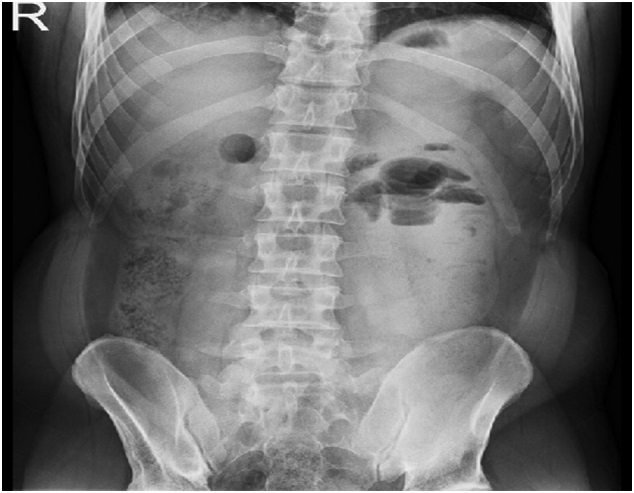
Native image of the abdomen (standing) – left paraduodenal hernia in a 39-year-old man.

*Radiograph of the abdomen*, *standing*: air and residual intestinal contents without pathological aeroliquid levels or intestinal distension. There is no accumulation of free air under the domes of the shield (Fig. 1).

*Abdominal ultrasound*: the liver is presented in a segmental intercostal approach due to meteorism, the segments shown are of appropriate echostructure, without dilatation of the bile ducts and convincing focal lesions. The gallbladder is of adequate size, of normal wall thickness, with no pathological intraluminal contents. The pancreas is visualized in part in the trunk area, which has a hyperechogenic structure of the parenchyma. The paraaortic area is not visualized due to flatulence. The spleen is of appropriate size, homogeneous echostructure of the parenchyma, without focal changes. The kidneys are of adequate location and size, of regular thickness and echostructure of the parenchyma, without signs of lithiasis and dilatation of the duct system. There is no free fluid in the abdomen and pelvis. The bladder is filled with a moderate amount of urine, in the analysis available part without endoluminal proliferates.

**Fig. 2:**
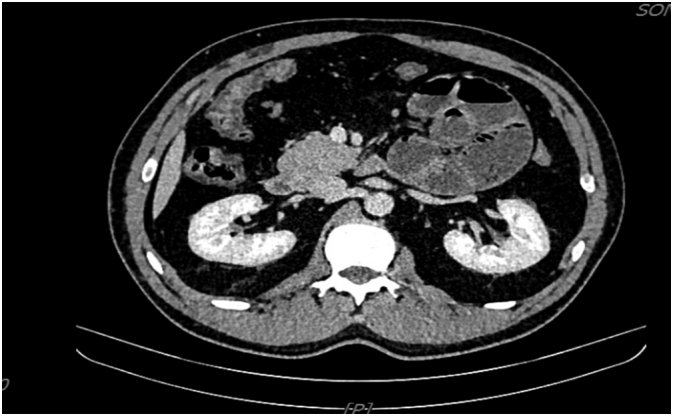
Axial contrast-enhanced abdominal computed tomography (CT) images of the left paraduodenal hernia in a 39-year-old man.

Since the laboratory findings, abdominal X-ray and abdominal ultrasound were inconspicuous, we decided on a diagnostic examination, contrast-enhanced abdominal computed tomography (CT), which showed the following result: in the left hemiabdomen, several convolutions of the jejunum of the distempered lumen 41 mm, filled with feces with abrupt conical narrowing, without clear cause, correspond to small bowel obstruction. A shorter segment of weaker wall opacification may indicate initial ischemic changes, with no signs of perforation. Intestinal convolutions aboral from the site of obstruction of empty, normal wall thickness. No pneumoperitoneum. Several reactive lymph nodes regionally, shorter in diameter up to 7 mm. Several tiny cysts of the liver and spleen without other focal or perfusion abnormalities. Bile ducts, gallbladder, pancreas, adrenal glands, kidneys, and small pelvic organs of normal size and density, without focal or perfusion abnormalities. There was no free fluid. Pulmonary bases without nodular lesions, consolidation and pleural effusion. Bone structures without signs of pathological bone remodeling (Fig. 2).

**Fig. 3:**
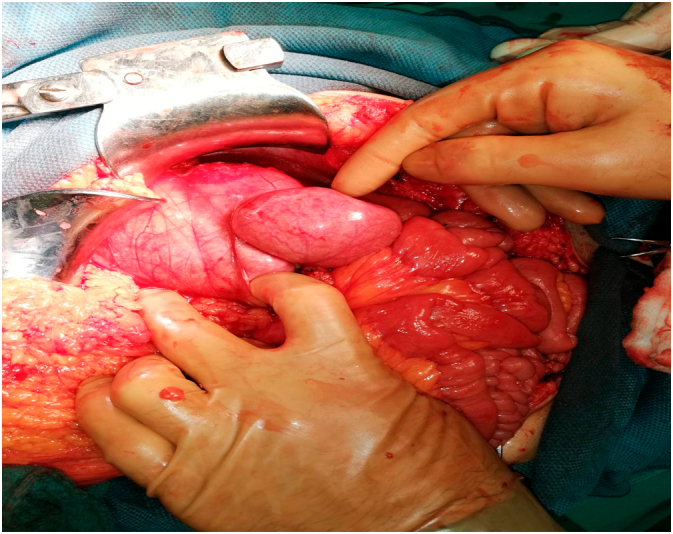
Intraoperative findings during open surgery for left paraduodenal hernia in a 39-year-old man. Captured jejunal loops.

**Fig. 4:**
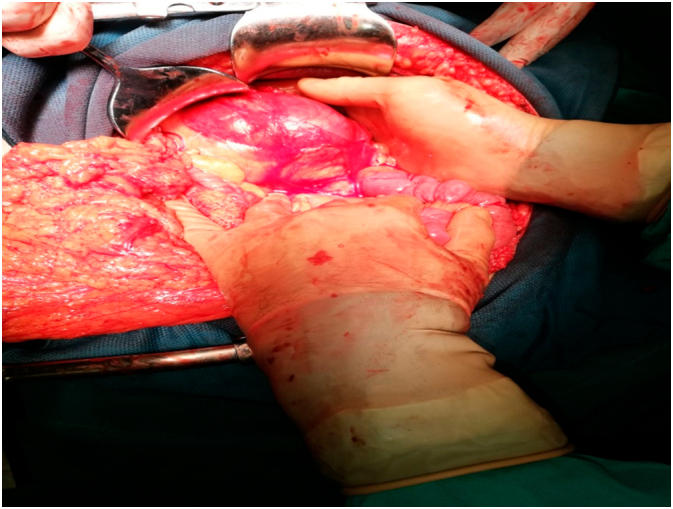
Intraoperative findings during open surgery for left paraduodenal hernia in a 39-year-old man. A conglomerate of small bowel convolutions within a hernia sac.

After preoperative preparation, the patient was placed under general anesthesia and surgery was performed. The procedure was performed by a general surgeon with 15 years of clinical experience. A laparotomy was performed through an upper midline incision. There was a minor serous effusion in the abdominal cavity. An inspection of the abdominal cavity followed, and a peritoneal sac was found with the proximal third of the jejunum twisted inside, located to the left of the fourth segment of the duodenum and the duodenojejunal junction. Collapsed loops of the small intestine were observed, aboral of the obstruction. With a meticulous preparation technique, the hernia sac was opened, the trapped jejunum loops were decompressed, the hernia sac was resected and the hernial opening was closed by sutures. Clamped jejunum loops showed signs of maintained vitality (normal color and bowel motility). This was followed by inspection and palpation of other structures of the abdominal cavity, and no other pathology was found. Rinsing of the abdominal cavity with warm saline and irrigation solution was performed, followed by drainage and closing of the wound in layers (Figs. 3 and 4). The postoperative course was normal. On the second postoperative day, the patient had a satisfactory local and general status and was transferred from the intensive care unit to the surgical department, with transition to oral nutrition. In the ward, he received oral nutrition with analgesic therapy as needed. On the fifth postoperative day, the patient was discharged with good general and local status. The wound healed primarily and sutures were removed on the eighth postoperative day. The patient was monitored via clinical examinations once a week for the first month after discharge, and then monthly. He was in good clinical condition and did not have any complaints in the clinical interview.

## 4 Discussion

LPDH represents herniation of the mesentery of the small intestine in the left paraduodenal or Landzert's fossa in the posteroinferior direction [11], [12]. Restrictions of the paraduodenal fossa consist of the fourth segment of the duodenum (right), the left branches of the middle colic artery (anteriorly), the posterior parietal attachment of the descending colon mesentery (posteriorly), and the inferior mesenteric vein [13], [14]. The cause of this opening is an embryonic disorder in terms of lack of connection of both peritoneal folds from the sixth to the eleventh week of gestation [7]. According to Schizas et al., the mean age of onset of paraduodenal hernia is 44.1 years (Schizas), and according to Muneer et al., it is between the ages of 40 and 60. It is three times more common in men than in women [4].

Clinical presentation of paraduodenal hernia is not unambiguous but ranges from asymptomatic to manifest (abdominal pain as the most common symptom, vomiting, nausea, symptoms of intestinal obstruction). Among the rarer symptoms that can be mentioned are secondary pancreatitis, biliary colic and palpable tumefaction in the upper left part of the abdomen. In many cases, the patient has non-specific symptoms which last for years. This case report concerns such a patient, a 39-year-old man. The greatest difficulty in management of paraduodenal hernias lies in their diagnosis, especially in the case of asymptomatic patients or non-specific symptoms of a chronic nature. In such cases, with a well-taken medical history, clinical examination of the patient, abdominal X-ray, abdominal ultrasound and laboratory tests and possible angiography of the upper mesenteric artery (displacement of jejunal arteries in the upper left quadrant of the abdomen), contrast CT scan of the abdomen plays a crucial role (accuracy 95%, sensitivity 95–100%), and in most cases confirms the diagnosis [15]. We must not forget that in a certain number of patients, definitive diagnosis is made during surgery or autopsy [9]. Definitive treatment of LPDH involves surgery, which can be performed laparoscopically or openly. The procedure involves releasing the intestinal loops from the hernia sac and repairing the defect by closing or widely opening the hernia orifice, whereby the hernia sac becomes a part of the peritoneal cavity. With the laparoscopic approach, early recovery is faster, but long-term results are similar [16], [17], [18], [19], [20], [21]. In the case of our patient, LPDH was easily reduced, so the primary closure of the hernia orifice with sutures was sufficient.

## 5 Conclusion

In this paper, a case of a 39-year-old male patient with LPDH with non-specific symptomatology was presented. Due to the intensification of abdominal pain, nausea and vomiting after meals as a result of intestinal obstruction, we approached a detailed diagnostic treatment of the patient and arrived at the above diagnosis. We performed open surgery, which confirmed the formation of LPDH, and we resolved it in the usual way, by releasing the trapped loops of the small intestine and closing the hernia orifice with sutures. Unrecognized LPDH leads to delayed surgery, which can cause intestinal perforation, peritonitis, and even death. Computed tomography of the abdomen helps us make an accurate diagnosis and perform timely surgery, which was the case with our patient.

## Sources of funding

We did not have any source of funding.

## Ethical approval

We did not use any personal date of patient included in this case report.

## Consent

Written informed consent was obtained from the patient for publication of this case report.

## Registration of research studies

N/A.

## Guarantor

Dragan Manojlović.

## CRediT authorship contribution statement

Dragan Manojlović: case report concept, writing the paper.

Nenad Čekić: Writing the paper.

Mario Palinkaš: data collection, writing the paper.

## Declaration of competing interest

There was not any conflict of interest.
